# Gestational-age-specific reference ranges for blood pressure in pregnancy: findings from a prospective cohort

**DOI:** 10.1097/HJH.0000000000000368

**Published:** 2014-12-04

**Authors:** Corrie Macdonald-Wallis, Richard J. Silverwood, Abigail Fraser, Scott M. Nelson, Kate Tilling, Debbie A. Lawlor, Bianca L. de Stavola

**Affiliations:** a MRC Integrative Epidemiology Unit at the University of Bristol; b School of Social and Community Medicine, University of Bristol, Bristol; c Centre for Statistical Methodology; d Department of Medical Statistics, London School of Hygiene and Tropical Medicine, London; e School of Medicine, University of Glasgow, Glasgow, UK

**Keywords:** Avon Longitudinal Study of Parents and Children, blood pressure, longitudinal, pregnancy, reference range

## Abstract

**Objective:**

Pregnancy is a period of considerable change in blood pressure, with an early pregnancy decrease followed by a late pregnancy rise. High blood pressure in pregnancy is associated with adverse perinatal outcomes for the mother and offspring. We aimed to define normal ranges of blood pressure across gestation.

**Methods:**

We used repeated antenatal blood pressure measurements [median (interquartile range) 10 (9–11) per woman] for 10 327 women. Multilevel models were used to derive longitudinal reference ranges for SBP and DBP from 12 to 40 weeks gestation for the whole cohort, for women with normal pregnancies (without essential hypertension or preeclampsia who delivered an appropriate-size-for-gestational age infant at term) and for subgroups of normal pregnancies defined by different levels of maternal prepregnancy BMI, smoking and parity.

**Results:**

In normal pregnancies, the mean (95% reference range) SBP and DBP for nulliparous women at 12 weeks gestation were 112.1 (88.6–135.5) and 65.4 (48.9–81.9) mmHg, and at 37 weeks were 116.0 (92.3–139.7) and 70.0 (52.2–87.9) mmHg, respectively. For every additional 10 mmHg of blood pressure at 12 weeks, normal ranges were 2–3 mmHg higher across gestation. Reference ranges for multiparous women were 1–2 mmHg lower throughout pregnancy. Stratified reference ranges were higher for women in higher prepregnancy BMI categories, and lower for smokers than for nonsmokers throughout pregnancy.

**Conclusion:**

Normal ranges for blood pressure vary with gestation age and by maternal subgroups. Whole population and stratified normograms could be used as a reference to identify abnormal trajectories.

## INTRODUCTION

Around 10% of women experience some form of hypertension during pregnancy [1,2], which may be preexisting or pregnancy-induced. These pregnancies carry a greater risk of a range of adverse perinatal outcomes, including maternal and foetal death [3–5], intrauterine growth restriction of the infant and preterm birth [6–8]. However, pregnancy is a period of substantial change in blood pressure, with an early pregnancy decrease followed by a steep rise in the latter half of pregnancy [9,10]. There is evidence that, even among women without preexisting hypertension or preeclampsia [11], a greater increase in blood pressure, and the maximum level reached, are also associated with reduced foetal growth [11,12].

Furthermore, the early-pregnancy level and the average change in blood pressure have been found to differ by maternal prepregnancy BMI, smoking and parity [10,13,14], even among women who then experienced a healthy birth outcome [10]. This would imply that different trajectories of blood pressure may be healthy for different subgroups of women. However, differences in blood pressure trajectories by maternal age and education were much smaller [10]. It is also plausible that the normal trajectory may depend on the initial level of blood pressure, as a greater increase is likely to be tolerable for a woman who begins pregnancy with a relatively low blood pressure. Establishing blood pressure reference ranges across pregnancy for different subgroups of women, who do not experience adverse health outcomes, may provide important information about what is normal at different gestational ages for different women. These normal ranges could be useful for the identification of women whose blood pressure is deviating from a healthy trajectory before they cross the high blood pressure threshold, and thus lead to earlier detection of women at risk. Despite the clear importance of blood pressure in pregnancy, to our knowledge, there are currently no normal reference ranges to aid clinical interpretation of the repeat antenatal monitoring that is routinely carried out.

Our aim was to develop reference ranges for SBP and DBP across pregnancy, firstly for all women regardless of pregnancy outcome and then for women who have healthy term pregnancies (without essential hypertension or preeclampsia, resulting in a live birth of an appropriate-size-for-gestational-age baby) in a large population-based prospective cohort. We also divided the group of women who had healthy term pregnancies into a low-risk and a high-risk group on the basis of maternal characteristics and fitted reference ranges that were stratified by parity, BMI, smoking and early-pregnancy blood pressure in order to allow for a different normal trajectory of blood pressure change for different subgroups of women.

## MATERIALS AND METHODS

The Avon Longitudinal Study of Parents and Children (ALSPAC) is a prospective cohort study investigating the health and development of children. The study has been described in full elsewhere [15] and the study website contains details of available data at http://www.bris.ac.uk/alspac/researchers/data-access/data-dictionary. In brief, pregnant women with an expected delivery date between 1 April 1991 and 31 December 1992 living in a defined area of Avon, South West England, were eligible for recruitment. Ethical approval for the study was obtained from the ALSPAC Law and Ethics Committee and from the National Health Service (NHS) local ethics committee. In total, 14 541 women were enrolled and 13 678 had a singleton pregnancy resulting in a live birth. We excluded multiple pregnancies, as these would have different patterns of blood pressure change and there were insufficient numbers to study these in detail. Thirteen thousand, four hundred and sixty-one of the women had data abstracted from obstetric records and 13 000 had at least one blood pressure measurement during pregnancy. Of these, 10 327 had complete data on all maternal characteristics.

### Obstetric measurements

All SBP and DBP measurements that were taken as part of routine antenatal care by midwives or obstetricians were abstracted from the women's obstetric records by six trained research midwives. There was no between-midwife variation in mean values of the data abstracted and error rates were consistently less than 1% in repeated data entry checks. These were single blood pressure measurements taken in the seated position using the appropriate cuff size, using Korotkoff phase V for DBP. The gestational age of measurement was derived from the date of measurement and the expected delivery date. Gestational age at delivery was derived from the expected delivery date and the date of birth. For most women, the expected delivery date was based on the last menstrual period date, while for a small proportion, this estimate was updated following an ultrasound scan. In the data abstracted from the clinical records, it was not recorded which few women had a scan or had their gestational age adjusted.

### Birth size

The child's birthweight was obtained from the birth notification. We defined small-for-gestational age (SGA) as below the 10th percentile of birthweight for gestational age and large-for-gestational age (LGA) as above the 90th percentile of birthweight for gestational age within this cohort by regressing birthweight on gestational age and extracting the residuals.

### Hypertension and diabetes before and during pregnancy

Using all available blood pressure measurements, preeclampsia was defined using the International Society for the Study of Hypertension in Pregnancy (ISSHP) definition as SBP at least 140 mmHg and/or DBP at least 90 mmHg as well as proteinuria of 1+ or more on urine dipstick testing on two occasions after 20 weeks gestation in a woman who was not known to be hypertensive prior to pregnancy [16]. Women who responded to a questionnaire administered during pregnancy that they had previously had hypertension outside of pregnancy were considered to have essential hypertension. Women who responded that they had previously had diabetes outside of pregnancy were classed as having existing diabetes, and for those without existing diabetes, any diagnosis of gestational diabetes was obtained from obstetric records.

### Other maternal characteristics

Maternal prepregnancy weight, height, parity and smoking status were obtained from questionnaires administered during pregnancy. Prepregnancy BMI was calculated as weight (kg)/height (m)^2^ and classed according to WHO definitions of underweight (<18.5 kg/m^2^), normal (18.5–24.9 kg/m^2^), overweight (25.0–29.9 kg/m^2^) and obese (≥30.0 kg/m^2^). A questionnaire at 18 weeks gestation asked about smoking status, which was categorized as ‘never during pregnancy’; ‘prepregnancy/first trimester’ for women who smoked immediately prior to pregnancy or in the first 3 months and then stopped; or ‘throughout’ for women who continued to smoke after the first 3 months.

### Definition of normal pregnancy

To restrict to normal pregnancies, we excluded 515 (5.0%) women who delivered preterm (<37 weeks gestation), 937 (9.1%) who had an SGA offspring, 1040 (10.1%) who had an LGA offspring, 369 (3.6%) with preexisting hypertension, 213 (2.1%) who developed preeclampsia, 38 (0.4%) with existing diabetes and 46 (0.4%) who developed gestational diabetes (total excluded = 2823). We did not exclude women who had gestational hypertension, as this would have removed the top end of the blood pressure distribution and biased reference ranges downwards. After these exclusions, there remained 7504 women who had a normal pregnancy.

### Statistical analysis

To develop longitudinal reference ranges for SBP and DBP across gestation, we used multilevel models with two levels: measurement occasions (level 1) within women (level 2). These models take into account that the repeated measurements of blood pressure within individuals are not independent, and allow for varying numbers and timings of measurements between women under a missing at random assumption [17,18].

Separate multilevel models were fitted with SBP and DBP as the outcome, respectively, and each had gestational age as the exposure, assuming normal variation in the population at each gestational age. Restricted cubic splines with knots at 11, 18, 30, 36 and 40 weeks gestation were used to describe the shape of the blood pressure trajectory over gestation. Full information is provided in online supplemental material (including the choice of knots). The baseline gestational age was set at 12 weeks, as this was the median time of the first antenatal measurement. In addition to gestational age, the models included other explanatory variables as described below, or were fitted separately on certain subgroups of women to allow for complexity in their trajectories. Ninety-five percent reference ranges for SBP and DBP from 12 to 40 weeks gestation were then estimated from each fitted model.

We first fitted reference ranges for all women who had complete data on all maternal characteristics, regardless of pregnancy outcome. We then restricted to women who had a normal pregnancy (see definition above). Finally, we divided the women who had a normal pregnancy into subgroups and fitted normal reference ranges for the different subgroups.

The subgroups of interest were defined by the cross-classification of BMI and smoking status. The ‘low-risk’ group comprised those who had normal prepregnancy BMI and did not smoke either immediately prior to or during pregnancy. The ‘high-risk’ group included women who were overweight/obese and who smoked either immediately prior to pregnancy or in the first trimester only or who smoked throughout pregnancy. Further reference ranges were produced by prepregnancy BMI category (including only those who never smoked) and by smoking status (including only those with a normal prepregnancy BMI). In order to predict blood pressure trajectories for the entire duration of pregnancy from a single blood pressure measure at 12 weeks, as would be available in a clinical setting, reference ranges conditional on such possible values were developed for the low-risk group (see supplemental material) [19]. In each of these analyses, separate models were fitted for nulliparous and multiparous women. A full list of the subgroups for whom reference ranges have been developed is given in Supplementary Table 1.

As a sensitivity analysis, we refitted the reference ranges in the low-risk group as defined when using the customized foetal growth reference of Gardosi and Francis [20] to define SGA and LGA. Reference ranges were not meaningfully different from those presented here.

We used MLwiN version 2.27 to fit the multilevel models that we ran through Stata using the runmlwin command [21]. The reference range figures were produced in R version 2.15.1. Stata version 12.1 (Stata Corp. College Station, Texas, USA) was used for all other analyses.

## RESULTS

The characteristics of women who had complete data for inclusion in reference ranges and those that were excluded from analysis due to missing maternal characteristic data are summarized in Table 1. Women who were included were generally similar to those who were excluded, although they were slightly more likely to be underweight. Women who were included who had normal pregnancies had lower SBP and DBP at 12 and 37 weeks gestation and were less likely to be overweight/obese, less likely to be nulliparous and less likely to smoke throughout pregnancy than women who were considered to have an abnormal pregnancy due to either a medical condition (essential hypertension, preeclampsia, existing or gestational diabetes *N* = 647) or an adverse perinatal outcome (preterm birth, SGA or LGA offspring; *N* = 2404).

**FIGURE 1:**
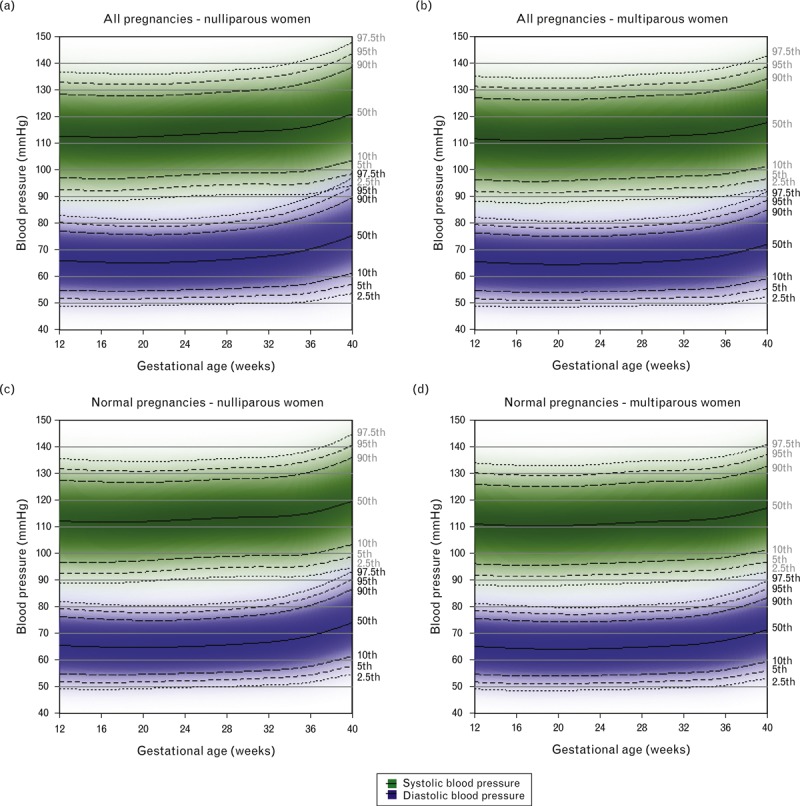
Reference ranges for SBP and DBP between 12 and 40 weeks gestation in the full cohort with complete data (nulliparous *N* = 4718; multiparous *N* = 5609) and in normal pregnancies only (nulliparous *N* = 3372; multiparous *N* = 4132). Centiles are labelled. A normal pregnancy was defined as one in which the woman did not have essential hypertension, preeclampsia, existing or gestational diabetes and delivered an appropriate-for-gestational-age sized infant at term.

Figure 1 shows the reference ranges for SBP and DBP between 12 and 40 weeks gestation for all women and for those women who had a normal pregnancy, by parity. The predicted values and 95% reference ranges at 12, 20 and 37 weeks gestation are summarized in Supplementary Table 2. The reference ranges for SBP and DBP decreased until mid-pregnancy in both nulliparous and multiparous women before rising until the end of pregnancy, although the decrease was more pronounced in multiparous women than in nulliparous women. The average timing of the nadir in SBP varied, occurring at 17 weeks in nulliparous women and 18 weeks in multiparous women in the whole cohort and also when restricting to normal pregnancies. The nadir in DBP occurred at around 19 weeks of gestation in nulliparous women and 20 weeks in multiparous women in the whole cohort and at around 20 weeks for both nulliparous and multiparous women when restricting to normal pregnancies. The 95% reference range for SBP was approximately 45–50 mmHg wide and for DBP was approximately 30–35 mmHg wide in the whole cohort, both widening slightly towards the end of gestation. Upper reference range limits were generally slightly lower across gestation in normal pregnancies than in the whole cohort but, as ranges were also slightly narrower, the lower reference range limits were similar in the whole cohort and in normal pregnancies.

### Reference ranges in subgroups of normal pregnancies

**FIGURE 2:**
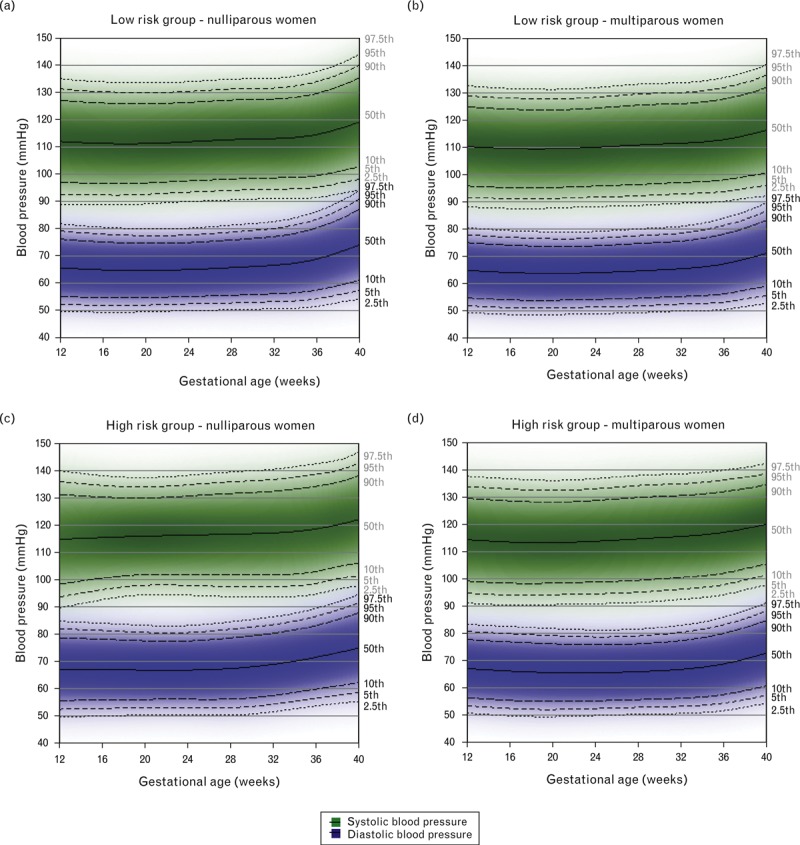
Reference ranges for systolic and diastolic blood pressure between 12 and 40 weeks gestation in low-risk (nulliparous *N* = 1832; multiparous *N* = 2193) and high-risk (nulliparous *N* = 205; multiparous *N* = 285) normal pregnancies. Centiles are labelled. The low-risk group included women who had a normal prepregnancy BMI and did not smoke either immediately prior to or during pregnancy. The high-risk group included overweight or obese women who smoked either immediately prior to pregnancy, in the first trimester or throughout pregnancy.

The reference ranges of SBP and DBP across gestation in the low-risk group (normal prepregnancy BMI; never smoked) and in the high-risk group (overweight/obese; smoked at any time either immediately prior to or during pregnancy) of women who had normal pregnancies are shown in Fig. 2. Supplementary Table 2 summarizes the predicted values and 95% reference ranges of SBP and DBP at 12, 20 and 37 weeks gestation. Reference ranges in the high-risk group were generally around 4 mmHg higher than in the low-risk group across pregnancy for both nulliparous and multiparous women. SBP for nulliparous women in the high-risk group did not show a mid-pregnancy dip, but increased throughout pregnancy.

**FIGURE 3:**
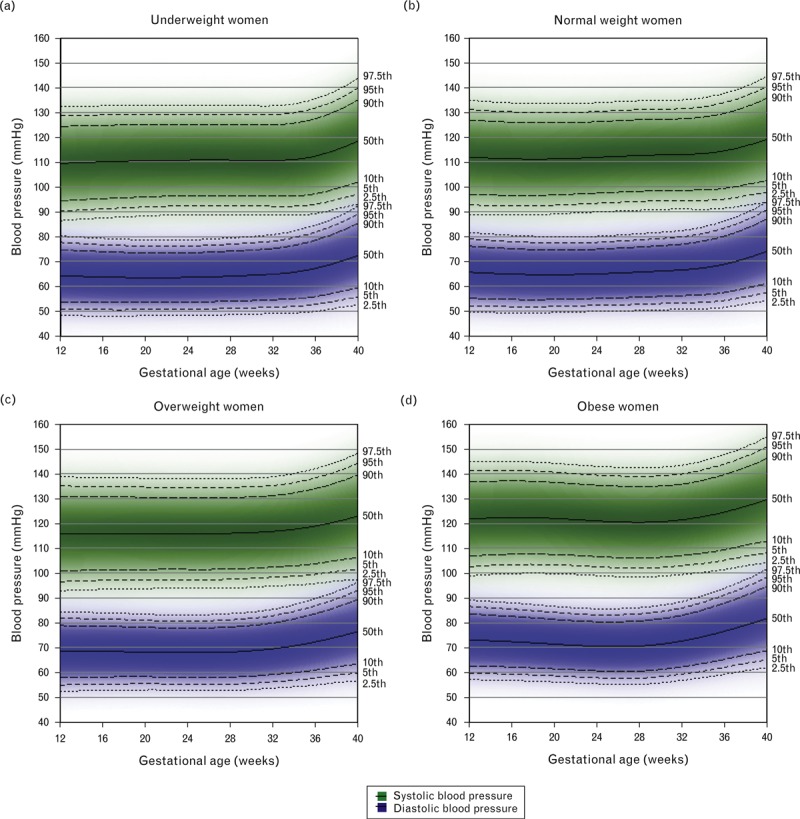
Reference ranges for SBP and DBP by maternal prepregnancy BMI category for nulliparous nonsmokers with normal pregnancies (*N* = 2270). Centiles are labelled.

Reference ranges stratified by maternal prepregnancy BMI are shown for nulliparous nonsmoking women in Fig. 3 and for multiparous women in Supplementary Figure 1. For both nulliparous and multiparous women, there was an increasing trend in the limits of the reference ranges with increasing prepregnancy BMI category at each gestational age. For example, for nulliparous women, upper and lower reference range limits of SBP were approximately 10 mmHg higher throughout pregnancy for obese than for normal weight women, and for DBP were approximately 7.5–8 mmHg higher (Supplementary Table 2). The shape of the blood pressure trajectory also differed by prepregnancy BMI category (Supplementary Figures 2 and 3), with obese women having a more distinct nadir in SBP and DBP than normal weight women in the nulliparous group.

**FIGURE 4:**
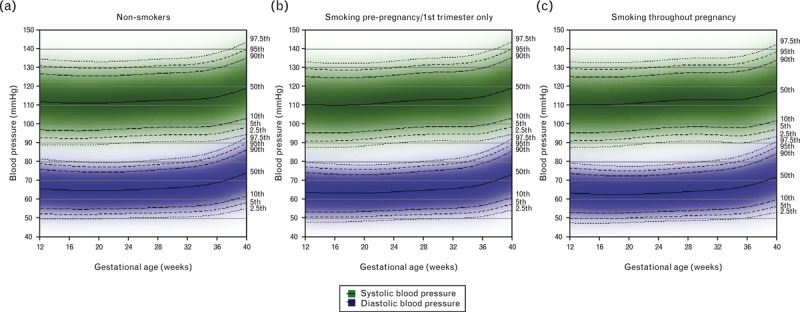
Reference ranges for SBP and DBP by maternal smoking during pregnancy for nulliparous normal-weight women with normal pregnancies (*N* = 2654). Centiles are labelled.

Reference ranges stratified by smoking during pregnancy are shown in Fig. 4 for nulliparous normal-weight women and Supplementary Figure 4 for multiparous women. SBP reference ranges were around 0–1.5 mmHg lower across pregnancy for women who smoked throughout pregnancy than for nonsmokers, while DBP reference ranges were around 1–2 mmHg lower (Supplementary Table 2). Those who smoked only prepregnancy/first trimester had 1–2 mmHg lower SBP and DBP reference ranges in early pregnancy than nonsmokers, but this difference attenuated towards the end of gestation. The average SBP and DBP trajectories by smoking status are shown in Supplementary Figures 5 and 6, respectively.

**FIGURE 5:**
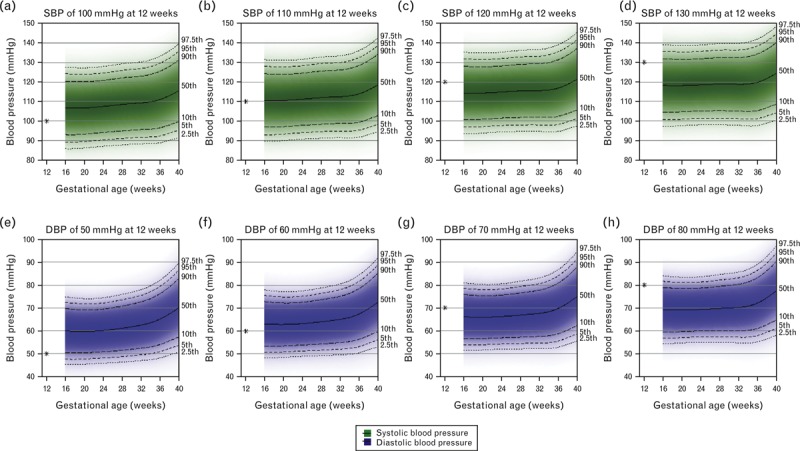
Reference ranges for SBP and DBP in pregnancy conditional on the level of blood pressure at 12 weeks gestation for nulliparous normal-weight nonsmoking women with normal pregnancies (*N* = 1832). Centiles are labelled. Note that in each of the plots, there is a star that corresponds to the value of SBP/DBP at 12 weeks.

Figure 5 shows the predicted reference ranges of SBP and DBP across pregnancy, conditional on different possible levels of blood pressure at 12 weeks gestation for nulliparous women. The equivalent reference ranges for multiparous women are shown in Supplementary Figure 7. For each additional 10 mmHg in SBP at 12 weeks, the reference ranges for SBP were predicted to be approximately 4 mmHg higher at 20 weeks and around 3 mmHg higher at 37 weeks gestation in both nulliparous and multiparous women (Supplementary Table 3). For each additional 10 mmHg in DBP at 12 weeks, the reference ranges for DBP were predicted to be around 3 mmHg higher at 20 weeks and around 2.5 mmHg higher at 37 weeks (Supplementary Table 3).

## DISCUSSION

We have derived reference ranges for blood pressure from 12 to 40 weeks gestation in a large population-based pregnancy cohort and compared these with reference ranges for women who had a normal pregnancy, without essential hypertension or preeclampsia, existing or gestational diabetes who delivered an appropriate-sized infant at term. We found that normal pregnancies had generally lower upper reference range limits and narrower reference ranges throughout pregnancy. We also observed differences in reference ranges between different subgroups of women with normal pregnancies based on prepregnancy BMI, smoking and parity and found that the expected normal trajectory of blood pressure differed by the blood pressure at the first antenatal visit (in this cohort at around 12 weeks gestation). To our knowledge, there are no existing longitudinal reference ranges for blood pressure in normal pregnancy. The shape of the average trajectory of blood pressure in pregnancy as decreasing until mid-pregnancy followed by an increase in late-pregnancy has been well described [9,22,23], and the addition of normal ranges to these average trajectories allows observed blood pressure measurements at any gestational age to be compared with expected limits.

This study benefits from its large size, repeated measurements of blood pressure and detailed information on maternal characteristics. The blood pressure measurements were collected during routine clinical practice, and thus will have greater measurement error than standardized measures. However, this means that the reference ranges are applicable to a clinical setting. The pregnancies occurred approximately 20 years ago and levels of obesity in the UK have increased over this period [24], although smoking prevalence has reduced [25]. However, the analyses are stratified by BMI and smoking and these changes are unlikely to have had a large effect on blood pressure levels within strata. The ALSPAC participants also differed slightly from the UK as a whole, being more likely to live in owner-occupied accommodation and to have a car and less likely to be nonwhite [15]. In the interests of space, we have not presented reference ranges for all possible subgroups of women, but have provided examples of low and high-risk groups and demonstrated how reference ranges differ with each of the maternal characteristics. This approach could be extended to develop customized blood pressure charts for all women based on prepregnancy BMI, smoking, parity and first blood pressure measurement; however, we did not have sufficient power to derive reference ranges for some of the smaller subgroups in the present study. The graphs presented here are illustrative and validation in other cohorts is required before these can be used in clinical practice.

The differences in normal ranges between obese and normal weight women of nearly 8–10 mmHg in SBP and nearly 6.5–8 mmHg in DBP at each gestational age are in line with the differences in average trajectories found in our previous study in this cohort with weaker exclusion criteria [10], in the Generation R study from the Netherlands [13] and in the U.S.-based Omega Study [26]. The two studies in other cohorts, however, included women with preeclampsia, preterm birth and SGA or LGA offspring. The magnitude of the differences between BMI categories may have important implications for risk-related levels of high blood pressure for women of different BMIs and at different gestational ages. For example, for nulliparous obese women, values of SBP over 140 mmHg were well within the 95% reference range across the whole of pregnancy, whereas for normal weight women, the 95% reference range remains below 135 mmHg for much of pregnancy.

Although smaller differences in blood pressure reference ranges were seen between smoking groups (up to 2 mmHg), they suggest that smoking status may be useful to include, along with prepregnancy BMI, in prediction models which use deviations from each woman's expected blood pressure trajectory to identify high-risk pregnancies. In our previous study, we found strong statistical evidence to support lower average blood pressure trajectories for smokers than for nonsmokers [10], and other cohorts have supported this finding for DBP, although findings relating to SBP have been conflicting [9,14,27]. It has been shown that blood pressure tracks moderately across pregnancy, with almost 50% of women remaining in the same third of blood pressure from the first trimester to the third trimester [23], which supports the differences that we found in the expected trajectories of blood pressure across pregnancy by blood pressure levels at 12 weeks.

The diagnostic criteria for hypertensive disorders of pregnancy (HDP) use the same blood pressure thresholds of 140/90 mmHg across the whole gestational period between 20 weeks and delivery and for all women, although nulliparous women, obese women and those with high prepregnancy blood pressure are considered to be at a high risk for HDP [28]. Our findings suggest that using different blood pressure thresholds for different maternal subgroups to identify women whose blood pressure has deviated from the normal trajectory may provide additional information about risk. Nevertheless, subgroups of women who are known to be at a higher risk of adverse perinatal outcomes should be considered as such, even with blood pressure within the normal range. Our reference ranges also show differences of around 5 mmHg in SBP and DBP between 20 weeks and delivery, suggesting that using different blood pressure thresholds at different gestational ages may improve the identification of women with abnormal trajectories.

There has been much research in the area of foetal growth charts [29–31], and more recently customized charts that are dependent on maternal characteristics [20]. Routine measurements of symphysis-fundal height and ultrasound scan measurements of foetal size may be compared against these charts and used to identify growth-restricted foetuses [32,33]. There may be potential for blood pressure charts in pregnancy to be used in conjunction with foetal growth charts to aid in the identification of pregnancies at risk of SGA infants, as high blood pressure and greater increases in blood pressure are associated with reduced offspring birthweight [11,12]. In addition, blood pressure reference ranges may help to identify women who are at risk of preeclampsia and delivering preterm. We have previously shown in this cohort [34] and others elsewhere [23] that the increase in blood pressure is steeper in preeclamptic than in normotensive pregnancies. Thus, preeclamptic women would be expected to deviate from the normal pattern of change, although the potential for deviations from the normal trajectory to be used in prediction of adverse outcomes remains to be assessed.

We conclude that the normal pattern of change in blood pressure across gestation, for a healthy term pregnancy resulting in an appropriate sized infant, differs by maternal subgroups. There may be potential to use whole population and stratified normal reference ranges such as these in screening for women with abnormal trajectories, which may be indicative of potential adverse pregnancy outcomes such as preeclampsia, preterm birth and SGA offspring. However, the value of this will require evaluation through the development and validation of prediction models.

## ACKNOWLEDGEMENTS

We are extremely grateful to all of the families who took part in this study, the midwives for their help in recruiting them and the whole ALSPAC team, which includes interviewers, computer and laboratory technicians, clerical workers, research scientists, volunteers, managers, receptionists and nurses.

This work was supported by the UK Wellcome Trust [grant number WT087997MA] and US National Institutes of Health [grant number R01 DK077659]. C.M.-W. and A.F. are funded by UK MRC research fellowships [grant numbers MR/J011932/1 and 0701594, respectively]. Core support for ALSPAC is provided by the UK Medical Research Council, the Wellcome Trust and the University of Bristol. C.M.W., K.T., A.F. and D.A.L. work in a unit that receives funds from the UK Medical Research Council (MRC) (MC_UU_12013/5 and MC_UU_12013/9).

### Conflicts of interest

There are no conflicts of interest.

## Supplementary Material

Supplemental Digital Content

## Figures and Tables

**TABLE 1:** Maternal characteristics of women who were excluded from analysis due to incomplete data and of women who were included by pregnancy type

	Pregnancies without complete data (*N* = 2673)		Pregnancies with complete data					
			All (*N* = 10 327)		Normal pregnancies (*N* = 7504)		Maternal medical condition or adverse pregnancy outcome (*N* = 2823)	
	*N* with data	Mean (SD) or %	*N*	Mean (SD) or %	*N*	Mean (SD) or %	*N*	Mean (SD) or %
Blood pressure								
SBP at 12 weeks (mmHg)	2673	112.1 (12.77)	10327	112.1 (12.12)	7504	111.5 (11.83)	2823	113.7 (12.72)
DBP at 12 weeks (mmHg)	2673	65.7 (9.06)	10327	65.7 (8.56)	7504	65.2 (8.30)	2823	66.8 (9.13)
SBP at 37 weeks (mmHg)	2673	116.0 (13.21)	10327	116.0 (12.92)	7504	115.0 (12.22)	2823	118.8 (14.22)
DBP at 37 weeks (mmHg)	2673	70.0 (10.24)	10327	69.9 (9.71)	7504	69.0 (9.06)	2823	72.4 (11.42)
Number of measures^a^ [median (IQR)]	2673	10 (8, 11)	10327	10 (9, 11)	7504	10 (9, 12)	2823	10 (8, 11)
Parity								
Nulliparous (%)	794	45.4	4718	45.7	3372	44.9	1346	47.7
Multiparous (%)	954	54.6	5609	54.3	4132	55.1	1477	52.3
Prepregnancy BMI								
Underweight (%)	35	7.1	514	5.0	379	5.1	135	4.8
Normal weight (%)	351	71.2	7665	74.2	5703	76.0	1962	69.5
Overweight (%)	77	15.6	1566	15.2	1074	14.3	492	17.4
Obese (%)	30	6.1	582	5.6	348	4.6	234	8.3
Smoking in pregnancy								
Never (%)	1044	55.8	7076	68.5	5176	69.0	1900	67.3
Prepregnancy/first trimester (%)	309	16.5	1373	13.3	1000	13.3	373	13.2
Throughout (%)	518	27.7	1878	18.2	1328	17.7	550	19.5

^a^This is the number of blood pressure measurements per woman after randomly selecting one measure per 2-week period of pregnancy for use in the multilevel models.
