# Supplementary material for: Gestational-age-specific reference ranges for blood pressure in pregnancy: findings from a prospective cohort
Source: J Hypertens. 2014 Dec 4;33(1):96–105. doi: 10.1097/HJH.0000000000000368 (PMC4255761; doi:10.1097/HJH.0000000000000368)
Supplement: Supplemental Digital Content [file jhype-33-96-s001.doc]

**Supplementary Methods**

Gestation was initially divided into two-week periods and, for women who had more than one blood pressure measurement available in any two-week period, one blood pressure measurement was selected at random per two-week period for each woman. This was to prevent women who had many measurements during pregnancy, who may represent less-healthy women, having a disproportionately high influence on the multilevel models.

Separate multilevel models with systolic blood pressure (SBP) and diastolic blood pressure (DBP) as outcomes were used to model the trajectory of change in SBP and DBP with gestational age. Several possible parameterisations of the shape of the curve were considered and the fit compared using the BIC and the difference between each individual’s predicted values and their actual blood pressure measurements across gestation. These included: fractional polynomial curves with up to two powers of gestational age, using the set of powers (-2, -1, -0.5, 0, 0.5, 1, 2, 3);[[1]](#_ENREF_1) linear splines with up to 3 knots and restricted cubic splines with up to 5 knots. Linear splines have been fitted previously to these data, and the best-fitting knot point locations were found to be at 18, 30 and 36 weeks gestation.[[2]](#_ENREF_2) The restricted cubic spline with 5 knots were fitted using knots selected by the percentile method of Harrell[[3]](#_ENREF_3) and also using the 3 knots found to be optimum for the linear splines (18, 30 and 36 weeks) for the middle 3 knots and the 5^th^ and 95^th^ percentiles of the data for the outer 2 knots (11 and 40 weeks). The fractional polynomial curve had a poorer fit compared with the linear or cubic splines. Linear splines and restricted cubic spline models with knots at 18, 30 and 36, and 11, 18, 30, 36 and 40 weeks respectively had the best fit to the data and fitted similarly. However, due to the smooth nature of the restricted cubic spline, reflecting the expected smooth change in blood pressure, this was selected and used for the normal reference ranges.

The models had two levels: measurement occasions (level1) within women (level2), reflecting the clustering of the data. Individual-level random effects were fitted for the intercept and for each of the cubic spline parameters, allowing the initial level and shape of the trajectory of change in blood pressure across gestation to vary between women. We allowed the within-individual variation in blood pressure to vary with gestational age as there was evidence of increasing variation in blood pressure as gestational age increased. Thus the model without covariates included took the following form:

$$\begin{matrix} y_{ij}=\beta_{0}+u_{0j}+(\beta_{1}+u_{1j})spline1_{ij}+(\beta_{2}+u_{2j})spline2_{ij}+(\beta_{3}+u_{3j})spline3_{ij}+(\beta_{4}+u{}_{4j})spline4_{ij}+ \\ e_{0ij}+e_{1ij}(GA_{ij}-12) \end{matrix}$$

$\left[\begin{matrix} u_{0j} \\ u_{1j} \\ u_{2j} \\ u_{3j} \\ u_{4j} \end{matrix}\right]\sim N\left(0, \Omega_{u}\right)$, $\left[\begin{matrix} e_{0ij} \\ e_{1ij} \end{matrix}\right]\sim N\left(0,\Omega_{e}\right)$

where, *y_ij_* is the value of the *i^th^* SBP or DBP measurement on the *j^th^* individual, *β_0_-β_4_* describe the average trajectory of change, *u_0j_-u_4j_* describe how the *j^th^* individual’s trajectory of SBP or DBP deviates from the average and *GA_ij_* is the gestational age in weeks of the *i^th^* measurement on the *j^th^* individual. It is centred at 12 weeks in order to set the intercept, β_0_, to represent blood pressure at 12 weeks. The e_0ij_ and e_1ij_ terms describe the deviation of the *i^th^* measurement of SBP or DBP on the *j^th^* individual from the individual’s trajectory. These are residual error terms.

The splines are defined as:[[3]](#_ENREF_3)

- 12

$$spline2_{ij}= \frac{(GA_{ij} -11)_{+}-(40-36)^{-1}\{(GA_{ij}-36)_{+}^{3}(40-11)-(GA_{ij}-40)_{+}^{3}(36-11)\}}{(40-11)^{2}}$$

$$spline3_{ij}= \frac{(GA_{ij} -18)_{+}-(40-36)^{-1}\{(GA_{ij}-36)_{+}^{3}(40-18)-(GA_{ij}-40)_{+}^{3}(36-18)\}}{(40-11)^{2}}$$

$$spline4_{ij}= \frac{(GA_{ij} -30)_{+}-(40-36)^{-1}\{(GA_{ij}-36)_{+}^{3}(40-30)-(GA_{ij}-40)_{+}^{3}(36-30)\}}{(40-11)^{2}}$$

$$where (x)_{+}=\left\{\begin{matrix} 0 if x\leq0 \\ x if x>0 \end{matrix}\right.$$

The variance of the individual blood pressure measurements at each gestational age was calculated as the sum of the between-individual-variance and the within-individual variance.[[4]](#_ENREF_4)

The between-individual variance at gestational age *g*, *V_bg_* =$\left[\begin{matrix} 1 \\ spline1_{g} \\ spline2_{g} \\ spline3_{g} \\ spline4_{g} \end{matrix}\right]^{T}\Omega_{u}\left[\begin{matrix} 1 \\ spline1_{g} \\ spline2_{g} \\ spline3_{g} \\ spline4_{g} \end{matrix}\right]$, where, for example, *spline1_g_* represents the value of the first cubic spline at gestational age *g*.

The within-individual variance at gestational age *g*, *V_wg_* = $\left[\begin{matrix} 1 \\ g-12 \end{matrix}\right]^{T}\Omega_{e}\left[\begin{matrix} 1 \\ g-12 \end{matrix}\right]$.

Therefore, the value of the 95^th^ percentile for the reference range at gestational age, *g*, is:

This gives the reference range for observed measures of blood pressure, rather than for an individual’s underlying blood pressure trajectory, as it includes both the between-individual and within-individual variances of blood pressure.

The fit of the model-predicted SBP and DBP across gestation to the observed SBP and DBP values in the nulliparous “low-risk” group (normal pre-pregnancy BMI and non-smoker) is shown in Tables 1 and 2 respectively, and in the multiparous “low-risk” group is shown in Tables 3 and 4. 90% of the predictions for SBP in each gestational period were generally within around 14 mmHg of the observed SBP values and 90% of predictions for DBP in each gestational period were generally within around 11 mmHg of the observed DBP values.

To produce reference ranges by pre-pregnancy BMI category, BMI was entered into the multilevel models as a categorical covariate (underweight, normal weight, overweight, obese) with normal weight as the reference category. A main effect term was included for BMI and also interactions with each of the spline variables. Reference ranges by smoking status were produced similarly, by entering smoking status as a categorical covariate (never smoked, smoked pre-pregnancy/1^st^ trimester, smoked throughout pregnancy), with never smoked as the reference category, as a main effect and also as interactions with each of the splines. The between and within-individual variances were calculated in the same way as for the unadjusted model. Separate multilevel models were fitted for nulliparous and multiparous women.

For the reference ranges which were conditional on SBP or DBP at 12 weeks gestation we used the unadjusted multilevel models as above, fitted only for the low-risk group (normal BMI; non-smokers) of women who had normal pregnancies. We obtained conditional predictions from this using the method described by Tilling et al[[4]](#_ENREF_4) and Pan and Goldstein.[[5]](#_ENREF_5) The covariance between the deviations from the predicted curve at 12 weeks and *g* weeks is *V_b12,g_*=$\left[\begin{matrix} 1 \\ spline1_{12} \\ spline2_{12} \\ spline3_{12} \\ spline4_{12} \end{matrix}\right]^{T}\Omega_{u}\left[\begin{matrix} 1 \\ spline1_{g} \\ spline2_{g} \\ spline3_{g} \\ spline4_{g} \end{matrix}\right]$

Therefore, the predicted SBP or DBP at gestational age *g*, conditional on the value at 12 weeks gestation is:

Where y_12_ is the observed value of the outcome (either SBP or DBP) at 12 weeks gestation, V_b12_ is the between-individual variance in the outcome at 12 weeks and V_w12_ is the within-individual variance. Thus, the deviation of the 12-week measurement from the average trajectory was combined with the multilevel model information to predict what trajectory would be seen for the rest of gestation. Conditional predictions were produced for hypothetical SBP values of 100, 110, 120 and 130 mmHg and DBP values of 50, 60, 70 and 80 mmHg at 12 weeks gestation.

The variance of this prediction is $(V_{bg}+V_{wg})-\frac{V_{b12,g}^{2}}{(V_{b12}+V_{w12})}$ and is used to calculate the reference ranges.

We tested the fit of the reference ranges conditional on the value of blood pressure at 12 weeks to the measurements in this cohort, by comparing predicted reference ranges conditional on each individual’s blood pressure measurement at (or within 3 weeks either side of) 12 weeks gestation against their actual blood pressure measurements from 16 weeks onwards. The 95% reference ranges for SBP conditional on blood pressure at 12 weeks contained 94.5% of actual blood pressure measurements from 16 weeks gestation onwards for nulliparous women and 94.8% of actual blood pressure measurements for multiparous women. The 95% reference ranges for DBP conditional on blood pressure at 12 weeks contained 95.2% of actual blood pressure measurements for nulliparous women, and 94.6% for multiparous women.

Please note, the following 4 tables are part of the Supplemental Methods and are not referred to in the main text. Thus, they have been labelled as Table X, rather than Web-Table X.

| Gestational period (weeks) | Number of measurements | Observed SBP  Mean (SD) | Predicted SBP  Mean (SD) | Observed – Predicted SBP  Mean (SD) | 90% Limits of Agreement^*^ |
| --- | --- | --- | --- | --- | --- |
| Up to 8 | 192 | 112.5 (12.83) | 113.5 (6.07) | -1.03 (9.21) | (-13.94, 13.91) |
| 9 - 10 | 420 | 111.3 (11.52) | 112.0 (5.96) | -0.73 (7.97) | (-14.56, 12.20) |
| 11 – 12 | 739 | 112.4 (11.37) | 112.5 (6.13) | -0.08 (8.33) | (-13.83, 13.95) |
| 13 – 14 | 817 | 112.4 (11.96) | 111.4 (6.49) | 1.04 (8.70) | (-12.76, 15.16) |
| 15 – 16 | 699 | 112.4 (11.77) | 111.7 (6.61) | 0.67 (8.34) | (-13.12, 14.42) |
| 17 – 18 | 1062 | 110.9 (12.01) | 111.3 (7.01) | -0.46 (8.13) | (-14.42, 13.24) |
| 19 – 20 | 729 | 110.8 (11.31) | 111.2 (6.93) | -0.38 (7.63) | (-12.68, 13.06) |
| 21 – 22 | 924 | 110.9 (11.16) | 111.3 (7.04) | -0.35 (7.36) | (-12.48, 11.69) |
| 23 – 24 | 761 | 111.9 (11.53) | 111.8 (7.30) | 0.13 (7.52) | (-11.80, 13.30) |
| 25 – 26 | 930 | 112.2 (11.12) | 112.0 (7.37) | 0.18 (7.01) | (-11.56, 11.37) |
| 27 – 28 | 962 | 112.8 (11.27) | 112.6 (7.17) | 0.20 (7.13) | (-10.89, 12.21) |
| 29 – 30 | 1343 | 112.8 (11.31) | 112.7 (7.35) | 0.05 (7.32) | (-11.30, 11.89) |
| 31 – 32 | 1452 | 112.9 (11.35) | 112.9 (7.15) | 0.02 (7.58) | (-12.02, 12.00) |
| 33 – 34 | 1588 | 112.8 (11.14) | 113.2 (6.94) | -0.44 (7.76) | (-12.32, 11.92) |
| 35 – 36 | 1551 | 114.2 (11.68) | 114.1 (7.15) | 0.12 (8.10) | (-12.74, 13.09) |
| 37 – 38 | 1954 | 115.8 (12.38) | 115.5 (7.32) | 0.34 (8.27) | (-11.66, 13.87) |
| 39 – 40 | 1649 | 117.4 (12.66) | 117.5 (7.51) | -0.17 (8.21) | (-13.11, 14.02) |
| 41+ | 851 | 119.4 (12.77) | 119.5 (7.78) | -0.12 (8.09) | (-13.43, 12.67) |

Table 1 Model fit for low-risk SBP model in nulliparous women

^*^ The range within which 90% of differences between the observed and model predicted values lie.

Table 2 Model fit for low-risk DBP model in nulliparous women

| Gestational period (weeks) | Number of measurements | Observed DBP  Mean (SD) | Predicted DBP  Mean (SD) | Observed – Predicted DBP  Mean (SD) | 90% Limits of Agreement^*^ |
| --- | --- | --- | --- | --- | --- |
| Up to 8 | 192 | 66.5 (9.15) | 67.0 (3.78) | -0.48 (7.38) | (-12.55, 9.73) |
| 9 - 10 | 420 | 65.2 (7.81) | 65.8 (3.53) | -0.64 (6.20) | (-10.74, 10.16) |
| 11 – 12 | 739 | 66.0 (8.23) | 65.8 (3.79) | 0.22 (6.46) | (-10.31, 10.66) |
| 13 – 14 | 817 | 65.7 (8.13) | 65.3 (3.83) | 0.42 (6.41) | (-10.19, 11.15) |
| 15 – 16 | 699 | 65.3 (7.98) | 65.1 (3.95) | 0.27 (6.13) | (-9.42, 10.56) |
| 17 – 18 | 1062 | 64.5 (8.11) | 64.6 (4.02) | -0.12 (6.07) | (-10.07, 10.19) |
| 19 – 20 | 729 | 64.5 (7.74) | 64.6 (4.09) | -0.18 (5.60) | (-9.74, 9.01) |
| 21 – 22 | 924 | 64.2 (7.67) | 64.6 (4.23) | -0.33 (5.55) | (-9.05, 8.77) |
| 23 – 24 | 761 | 65.2 (7.98) | 64.9 (4.20) | 0.31 (5.78) | (-9.68, 9.45) |
| 25 – 26 | 930 | 65.0 (7.73) | 65.0 (4.33) | 0.03 (5.61) | (-8.94, 9.39) |
| 27 – 28 | 962 | 65.4 (8.07) | 65.5 (4.56) | -0.10 (5.47) | (-8.72, 8.82) |
| 29 – 30 | 1343 | 66.0 (7.77) | 65.8 (4.52) | 0.16 (5.46) | (-8.44, 9.20) |
| 31 – 32 | 1452 | 66.1 (7.93) | 66.2 (4.62) | -0.13 (5.67) | (-9.63, 8.90) |
| 33 – 34 | 1588 | 66.7 (8.29) | 66.8 (4.66) | -0.12 (6.02) | (-9.29, 9.54) |
| 35 – 36 | 1551 | 68.2 (8.64) | 68.0 (5.08) | 0.16 (6.00) | (-9.47, 10.58) |
| 37 – 38 | 1954 | 70.0 (9.17) | 69.9 (5.34) | 0.13 (6.22) | (-9.99, 10.48) |
| 39 – 40 | 1649 | 72.0 (9.89) | 72.3 (5.67) | -0.31 (6.41) | (-10.23, 10.56) |
| 41+ | 851 | 74.6 (10.11) | 74.4 (6.16) | 0.25 (6.35) | (-10.04, 10.52) |

^*^ The range within which 90% of differences between the observed and model predicted values lie.

Table 3 Model fit for low-risk SBP model in multiparous women

| Gestational period (weeks) | Number of measurements | Observed SBP  Mean (SD) | Predicted SBP  Mean (SD) | Observed – Predicted SBP  Mean (SD) | 90% Limits of Agreement^*^ |
| --- | --- | --- | --- | --- | --- |
| Up to 8 | 215 | 110.7 (10.35) | 111.9 (5.37) | -1.18 (7.73) | (-14.12, 11.20) |
| 9 - 10 | 505 | 110.3 (11.62) | 110.5 (5.53) | -0.16 (8.36) | (-13.52, 12.39) |
| 11 – 12 | 821 | 110.6 (11.34) | 110.5 (6.01) | 0.08 (8.12) | (-13.45, 13.66) |
| 13 – 14 | 985 | 110.8 (11.58) | 109.9 (5.96) | 0.89 (8.50) | (-12.79, 15.55) |
| 15 – 16 | 839 | 109.5 (11.51) | 109.7 (6.24) | -0.19 (7.92) | (-12.98, 13.10) |
| 17 – 18 | 1257 | 108.9 (11.32) | 109.5 (6.39) | -0.59 (7.95) | (-13.89, 12.92) |
| 19 – 20 | 858 | 109.3 (10.78) | 109.3 (6.28) | -0.01 (7.66) | (-12.48, 12.39) |
| 21 – 22 | 1062 | 109.8 (11.48) | 109.6 (6.95) | 0.25 (7.49) | (-12.29, 11.77) |
| 23 – 24 | 916 | 110.3 (10.55) | 110.1 (6.73) | 0.22 (7.28) | (-12.30, 11.27) |
| 25 – 26 | 1080 | 110.2 (11.62) | 110.4 (7.11) | -0.19 (7.53) | (-12.61, 12.55) |
| 27 – 28 | 1101 | 110.7 (11.17) | 110.6 (7.14) | 0.15 (7.23) | (-10.98, 12.20) |
| 29 – 30 | 1569 | 111.0 (11.19) | 111.1 (7.05) | -0.13 (7.12) | (-11.75, 11.43) |
| 31 – 32 | 1642 | 111.4 (11.29) | 111.4 (7.05) | 0.01 (7.47) | (-11.49, 12.27) |
| 33 – 34 | 1839 | 111.7 (11.42) | 111.6 (6.98) | 0.13 (7.87) | (-12.15, 13.64) |
| 35 – 36 | 1805 | 112.3 (11.28) | 112.4 (6.91) | -0.05 (7.73) | (-12.48, 12.01) |
| 37 – 38 | 2213 | 113.5 (11.39) | 113.5 (6.73) | -0.02 (7.68) | (-12.26, 13.20) |
| 39 – 40 | 2015 | 115.0 (12.12) | 115.1 (7.00) | -0.09 (7.91) | (-12.50, 13.20) |
| 41+ | 821 | 116.6 (11.90) | 116.5 (6.78) | 0.15 (8.01) | (-11.79, 13.85) |

^*^ The range within which 90% of differences between the observed and model predicted values lie.

Table 4 Model fit for low-risk DBP model in multiparous women

| Gestational period (weeks) | Number of measurements | Observed DBP  Mean (SD) | Predicted DBP  Mean (SD) | Observed – Predicted DBP  Mean (SD) | 90% Limits of Agreement^*^ |
| --- | --- | --- | --- | --- | --- |
| Up to 8 | 215 | 65.8 (7.52) | 66.1 (3.19) | -0.30 (6.39) | (-10.05, 11.76) |
| 9 - 10 | 505 | 65.0 (8.60) | 65.3 (3.43) | -0.25 (6.55) | (-10.65, 10.89) |
| 11 – 12 | 821 | 65.2 (7.87) | 64.9 (3.61) | 0.32 (6.17) | (-9.77, 10.57) |
| 13 – 14 | 985 | 64.6 (7.69) | 64.4 (3.64) | 0.16 (6.12) | (-10.27, 10.30) |
| 15 – 16 | 839 | 64.0 (7.95) | 64.1 (3.90) | -0.08 (5.96) | (-9.50, 10.30) |
| 17 – 18 | 1257 | 63.4 (7.90) | 63.7 (4.13) | -0.37 (5.83) | (-10.06, 9.42) |
| 19 – 20 | 858 | 63.6 (7.82) | 63.6 (4.10) | 0.06 (5.63) | (-8.94, 9.41) |
| 21 – 22 | 1062 | 63.8 (7.81) | 63.7 (4.35) | 0.10 (5.48) | (-8.62, 9.34) |
| 23 – 24 | 916 | 64.3 (7.64) | 63.9 (4.22) | 0.33 (5.60) | (-8.88, 9.12) |
| 25 – 26 | 1080 | 63.9 (7.84) | 64.0 (4.40) | -0.07 (5.54) | (-8.66, 9.30) |
| 27 – 28 | 1101 | 64.4 (7.84) | 64.3 (4.41) | 0.04 (5.33) | (-8.38, 9.33) |
| 29 – 30 | 1569 | 64.7 (7.83) | 64.7 (4.32) | -0.05 (5.46) | (-8.88, 9.05) |
| 31 – 32 | 1642 | 65.1 (7.75) | 65.1 (4.42) | -0.04 (5.53) | (-8.99, 8.73) |
| 33 – 34 | 1839 | 65.5 (7.89) | 65.6 (4.45) | -0.08 (5.69) | (-9.06, 9.50) |
| 35 – 36 | 1805 | 66.6 (8.41) | 66.5 (4.55) | 0.07 (5.98) | (-9.29, 10.39) |
| 37 – 38 | 2213 | 68.1 (8.82) | 68.0 (4.75) | 0.11 (6.14) | (-9.40, 10.47) |
| 39 – 40 | 2015 | 69.6 (9.22) | 69.7 (4.92) | -0.15 (6.36) | (-10.13, 10.47) |
| 41+ | 821 | 71.6 (9.66) | 71.5 (5.11) | 0.09 (6.47) | (-9.91, 10.90) |

^*^ The range within which 90% of differences between the observed and model predicted values lie.

**Reference List**

1. Royston P, Altman DG. Regression using fractional polynomials of continuous covariates - parsimonious parametric modeling. *J Roy Stat Soc C-App*. 1994;43:429-467

2. Macdonald-Wallis C, Tilling K, Fraser A, Nelson SM, Lawlor DA. Established pre-eclampsia risk factors are related to patterns of blood pressure change in normal term pregnancy: Findings from the Avon Longitudinal Study of Parents and Children (ALSPAC). *J Hypertens*. 2011;29:1703-1711

3. Harrell FE, Jr. *Regression modeling strategies: With applications to linear models, logistic regression and survival analysis.* New York: Springer; 2001.

4. Tilling K, Sterne JAC, Wolfe CDA. Multilevel growth curve models with covariate effects: Application to recovery after stroke. *Stat Med*. 2001;20:685-704

5. Pan HQ, Goldstein H. Multi-level models for longitudinal growth norms. *Stat Med*. 1997;16:2665-2678

|  | **Reference Range Group** | **Parity** | **Pre-pregnancy BMI** | **Smoking during pregnancy** | **Blood pressure at 12 weeks (mmHg)** |
| --- | --- | --- | --- | --- | --- |
| **All pregnancies** (Figure 1) | | Nulliparous | **-** | **-** | - |
|  |  | Multiparous | **-** | **-** | - |
| **Normal pregnancies** | **All normal pregnancies** (Figure 1) | Nulliparous | **-** | **-** | - |
|  |  | Multiparous | **-** | **-** | - |
|  | **Low-risk women**  (Figure 2) | Nulliparous | Normal weight | Never smoked | - |
|  |  | Multiparous | Normal weight | Never smoked | - |
|  | **High-risk women**  (Figure 2) | Nulliparous | Overweight/Obese | Any smoking | - |
|  |  | Multiparous | Overweight/Obese | Any smoking | - |
|  | **Reference ranges by pre-pregnancy BMI category**  (Figure 3 and Web-Figure 1) | Nulliparous | Underweight | Never smoked | - |
|  |  | Nulliparous | Normal weight | Never smoked | - |
|  |  | Nulliparous | Overweight | Never smoked | - |
|  |  | Nulliparous | Obese | Never smoked | - |
|  |  | Multiparous | Underweight | Never smoked | - |
|  |  | Multiparous | Normal weight | Never smoked | - |
|  |  | Multiparous | Overweight | Never smoked | - |
|  |  | Multiparous | Obese | Never smoked | - |
|  | **Reference ranges by smoking during pregnancy**  (Figure 4 and Web-Figure 4) | Nulliparous | Normal weight | Never smoked | - |
|  |  | Nulliparous | Normal weight | Pre-pregnancy/1^st^ trimester only | - |
|  |  | Nulliparous | Normal weight | Throughout | - |
|  |  | Multiparous | Normal weight | Never smoked | - |
|  |  | Multiparous | Normal weight | Pre-pregnancy/1^st^ trimester only | - |
|  |  | Multiparous | Normal weight | Throughout | - |
|  | **Reference ranges conditional on blood pressure at 12 weeks**  (Figure 5 and Web-Figure 7) | Nulliparous | Normal weight | Never smoked | SBP = 100 at 12 weeks |
|  |  | Nulliparous | Normal weight | Never smoked | SBP = 110 at 12 weeks |
|  |  | Nulliparous | Normal weight | Never smoked | SBP = 120 at 12 weeks |
|  |  | Nulliparous | Normal weight | Never smoked | SBP = 130 at 12 weeks |
|  |  | Nulliparous | Normal weight | Never smoked | DBP = 50 at 12 weeks |
|  |  | Nulliparous | Normal weight | Never smoked | DBP = 60 at 12 weeks |
|  |  | Nulliparous | Normal weight | Never smoked | DBP = 70 at 12 weeks |
|  |  | Nulliparous | Normal weight | Never smoked | DBP = 80 at 12 weeks |
|  |  | Multiparous | Normal weight | Never smoked | SBP = 100 at 12 weeks |
|  |  | Multiparous | Normal weight | Never smoked | SBP = 110 at 12 weeks |
|  |  | Multiparous | Normal weight | Never smoked | SBP = 120 at 12 weeks |
|  |  | Multiparous | Normal weight | Never smoked | SBP = 130 at 12 weeks |
|  |  | Multiparous | Normal weight | Never smoked | DBP = 50 at 12 weeks |
|  |  | Multiparous | Normal weight | Never smoked | DBP = 60 at 12 weeks |
|  |  | Multiparous | Normal weight | Never smoked | DBP = 70 at 12 weeks |
|  |  | Multiparous | Normal weight | Never smoked | DBP = 80 at 12 weeks |

Web-Table List of all subgroups of women for whom reference ranges have been developed^*^

^*^ Each row represents a subgroup of women for whom a reference range is presented in this paper. Cells marked with – indicate that this characteristic was not part of the criteria for defining the subgroup for this particular reference range

Web-Table Mean and 95% reference ranges for systolic and diastolic blood pressure at three gestational ages across pregnancy for different subgroups of women from multilevel models

| **Maternal Subgroup** | | | | **N** | **Mean (95% reference range)** | | | | | |
| --- | --- | --- | --- | --- | --- | --- | --- | --- | --- | --- |
|  |  |  |  |  | **SBP at 12 weeks** | **SBP at 20 weeks** | **SBP at 37 weeks** | **DBP at 12 weeks** | **DBP at 20 weeks** | **DBP at 37 weeks** |
| **All women**  (Figure 1) | | Nulliparous | | 4718 | 112.7  (88.6, 136.8) | 112.4  (88.9, 135.9) | 117.2  (91.6, 142.8) | 65.9  (48.9, 82.9) | 65.1  (48.9, 81.2) | 71.2  (51.3, 91.2) |
|  |  | Multiparous | | 5609 | 111.6  (88.2, 135.0) | 111.0  (87.7, 134.3) | 115.0  (90.6, 139.3) | 65.5  (48.9, 82.1) | 64.4  (48.4, 80.5) | 68.8  (50.3, 87.2) |
| **Normal pregnancies^*^** | **All normal pregnancies**  (Figure 1) | Nulliparous | | 3372 | 112.1  (88.6, 135.5) | 111.9  (89.4, 134.4) | 116.0  (92.3, 139.7) | 65.4  (48.9, 81.9) | 64.6  (49.2, 80.1) | 70.0  (52.2, 87.9) |
|  |  | Multiparous | | 4132 | 111.0  (88.1, 133.9) | 110.3  (87.8, 132.8) | 114.2  (91.0, 137.4) | 65.1  (49.0, 81.1) | 64.0  (48.4, 79.6) | 68.2  (51.0, 85.3) |
|  | **Low-risk (normal BMI, non-smokers)**  (Figure 2) | Nulliparous | | 1832 | 112.0  (88.9, 135.0) | 111.3  (89.0, 133.5) | 115.4  (92.0, 138.8) | 65.6  (49.5, 81.7) | 64.6  (49.4, 79.8) | 69.9  (52.0, 87.8) |
|  |  | Multiparous | | 2193 | 110.2  (88.0, 132.5) | 109.4  (87.6, 131.2) | 113.5  (90.6, 136.4) | 64.7  (49.2, 80.3) | 63.6  (48.4, 78.8) | 67.9  (50.8, 85.0) |
|  | **High-risk (overweight/ obese, any smoking)**  (Figure 2) | Nulliparous | | 205 | 114.7  (89.4, 139.9) | 115.9  (94.3, 137.4) | 119.2  (95.5, 143.0) | 67.0  (49.4, 84.7) | 66.7  (50.2, 83.1) | 72.2  (54.0, 90.4) |
|  |  | Multiparous | | 285 | 114.3  (91.0, 137.6) | 113.2  (90.4, 136.0) | 117.6  (94.9, 140.3) | 66.9  (50.6, 83.3) | 65.4  (49.3, 81.6) | 69.5  (52.1, 86.9) |
|  | **By BMI (non-smokers only)** | Nulliparous  (Figure 3) | Underweight | 97 | 109.5  (86.4, 132.5) | 110.6  (88.3, 132.8) | 113.6  (90.1, 137.2) | 64.2  (48.1, 80.3) | 63.4  (48.2, 78.6) | 68.3  (50.5, 86.2) |
|  |  |  | Normal | 1832 | 112.0  (89.0, 135.0) | 111.3  (89.0, 133.5) | 115.4  (91.9, 138.9) | 65.6  (49.5, 81.6) | 64.6  (49.4, 79.8) | 69.9  (52.1, 87.7) |
|  |  |  | Overweight | 275 | 115.9  (92.8, 138.9) | 116.0  (93.8, 138.2) | 119.8  (96.3, 143.3) | 68.4  (52.3, 84.4) | 68.2  (53.0, 83.4) | 73.2  (55.4, 91.1) |
|  |  |  | Obese | 66 | 121.9  (98.8, 144.9) | 122.0  (99.8, 144.3) | 125.7  (102.2, 149.3) | 73.1  (57.0, 89.2) | 71.4  (56.2, 86.6) | 77.8  (60.0, 95.6) |

| **Maternal Subgroup** | | | | **N** | **Mean (95% reference range)** | | | | | |
| --- | --- | --- | --- | --- | --- | --- | --- | --- | --- | --- |
|  |  |  |  |  | **SBP at 12 weeks** | **SBP at 20 weeks** | **SBP at 37 weeks** | **DBP at 12 weeks** | **DBP at 20 weeks** | **DBP at 37 weeks** |
| **Normal pregnancies^*^** | **By BMI (non-smokers only)** | Multiparous  (Web-Figure 1) | Underweight | 122 | 107.9  (85.4, 130.4) | 106.4  (84.4, 128.5) | 110.3  (87.4, 133.2) | 64.0  (48.4, 79.6) | 62.4  (47.3, 77.6) | 66.2  (49.1, 83.4) |
|  |  |  | Normal | 2193 | 110.2  (87.7, 132.7) | 109.4  (87.4, 131.4) | 113.5  (90.6, 136.4) | 64.7  (49.1, 80.3) | 63.6  (48.5, 78.8) | 67.9  (50.8, 85.1) |
|  |  |  | Overweight | 430 | 115.1  (92.6, 137.5) | 114.2  (92.2, 136.2) | 117.0  (94.1, 139.9) | 67.8  (52.2, 83.4) | 67.1  (52.0, 82.3) | 70.3  (53.1, 87.5) |
|  |  |  | Obese | 161 | 117.9  (95.4, 140.4) | 118.1  (96.0, 140.1) | 121.4  (98.5, 144.3) | 70.7  (55.1, 86.3) | 69.7  (54.6, 84.9) | 74.3  (57.2, 91.5) |
|  | **By smoking in pregnancy (normal weight women only)** | Nulliparous  (Figure 4) | Never smoked | 1832 | 112.0  (89.1, 134.9) | 111.3  (89.2, 133.3) | 115.4  (91.9, 138.9) | 65.6  (49.6, 81.5) | 64.6  (49.6, 79.6) | 69.9  (52.5, 87.2) |
|  |  |  | 1^st^ trimester | 443 | 110.4  (87.5, 133.3) | 110.5  (88.4, 132.6) | 115.5  (92.0, 139.0) | 63.8  (47.9, 79.8) | 63.3  (48.3, 78.3) | 69.0  (51.6, 86.3) |
|  |  |  | Throughout | 379 | 110.4  (87.5, 133.3) | 110.9  (88.8, 133.0) | 114.4  (90.9, 137.9) | 63.3  (47.3, 79.2) | 62.6  (47.6, 77.6) | 68.1  (50.7, 85.5) |
|  |  | Multiparous  (Web-Figure 4) | Never smoked | 2193 | 110.2  (88.1, 132.4) | 109.4  (87.6, 131.2) | 113.5  (90.5, 136.4) | 64.7  (49.1, 80.3) | 63.6  (48.5, 78.7) | 67.9  (51.1, 84.7) |
|  |  |  | 1^st^ trimester | 302 | 109.0  (86.8, 131.1) | 108.6  (86.8, 130.3) | 112.6  (89.6, 135.5) | 63.4  (47.8, 79.0) | 62.7  (47.6, 77.8) | 67.1  (50.2, 83.9) |
|  |  |  | Throughout | 554 | 109.8  (87.6, 131.9) | 109.3  (87.5, 131.1) | 113.3  (90.3, 136.2) | 63.3  (47.7, 78.9) | 62.3  (47.2, 77.4) | 66.4  (49.5, 83.2) |

^*^ Normal pregnancies are defined as those where the woman did not have essential hypertension or preeclampsia, pre-existing or gestational diabetes and the pregnancy resulted and gave birth to an appropriate-size-for-gestational-age baby at term

|  | Error prone measurement of SBP at 12 weeks (mmHg) | Mean (95% reference range) SBP prediction | | Error prone measurement of DBP at 12 weeks (mmHg) | Mean (95% reference range) DBP prediction | |
| --- | --- | --- | --- | --- | --- | --- |
|  |  | 20 weeks | 37 weeks |  | 20 weeks | 37 weeks |
| Nulliparous women  (Figure 5) | 100 | 106.7  (86.2, 127.2) | 111.9  (89.5, 134.4) | 50 | 59.7  (45.4, 74.1) | 66.2  (48.7, 83.7) |
|  | 110 | 110.5  (90.0, 131.0) | 114.8  (92.4, 137.3) | 60 | 62.9  (48.5, 77.2) | 68.6  (51.1, 86.0) |
|  | 120 | 114.3  (93.8, 134.8) | 117.8  (95.3, 140.2) | 70 | 66.0  (51.6, 80.3) | 70.9  (53.5, 88.4) |
|  | 130 | 118.1  (97.6, 138.6) | 120.7  (98.2, 143.1) | 80 | 69.1  (54.7, 83.4) | 73.3  (55.8, 90.8) |
| Multiparous women  (Web-  Figure 7) | 100 | 105.6  (85.4, 125.8) | 110.5  (88.5, 132.5) | 50 | 59.0  (44.7, 73.4) | 64.1  (47.5, 80.6) |
|  | 110 | 109.3  (89.1, 129.5) | 113.4  (91.4, 135.4) | 60 | 62.1  (47.8, 76.5) | 66.7  (50.1, 83.3) |
|  | 120 | 113.0  (92.8, 133.2) | 116.3  (94.3, 138.3) | 70 | 65.2  (50.9, 79.6) | 69.3  (52.7, 85.9) |
|  | 130 | 116.7  (96.5, 136.9) | 119.1  (97.1, 141.1) | 80 | 68.4  (54.0, 82.7) | 71.9  (55.3, 88.5) |

Web-Table Mean and 95% reference ranges for predicted systolic and diastolic blood pressure at 20 weeks and 37 weeks in normal pregnancies for normal weight non-smoking women conditional on hypothetical measurements of SBP and DBP at 12 weeks gestation from multilevel models

Web-Figure Reference ranges for systolic and diastolic blood pressure by maternal pre-pregnancy BMI category for multiparous non-smokers with normal pregnancies (N=2906)


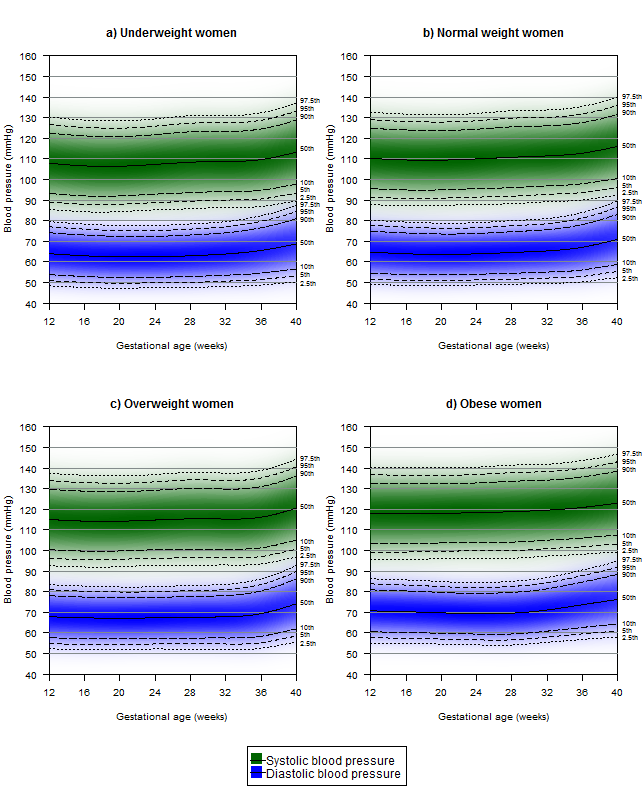


Web-Figure Average trajectories of systolic blood pressure by maternal pre-pregnancy BMI category in a) nulliparous and b) multiparous non-smoking women with normal pregnancies

**
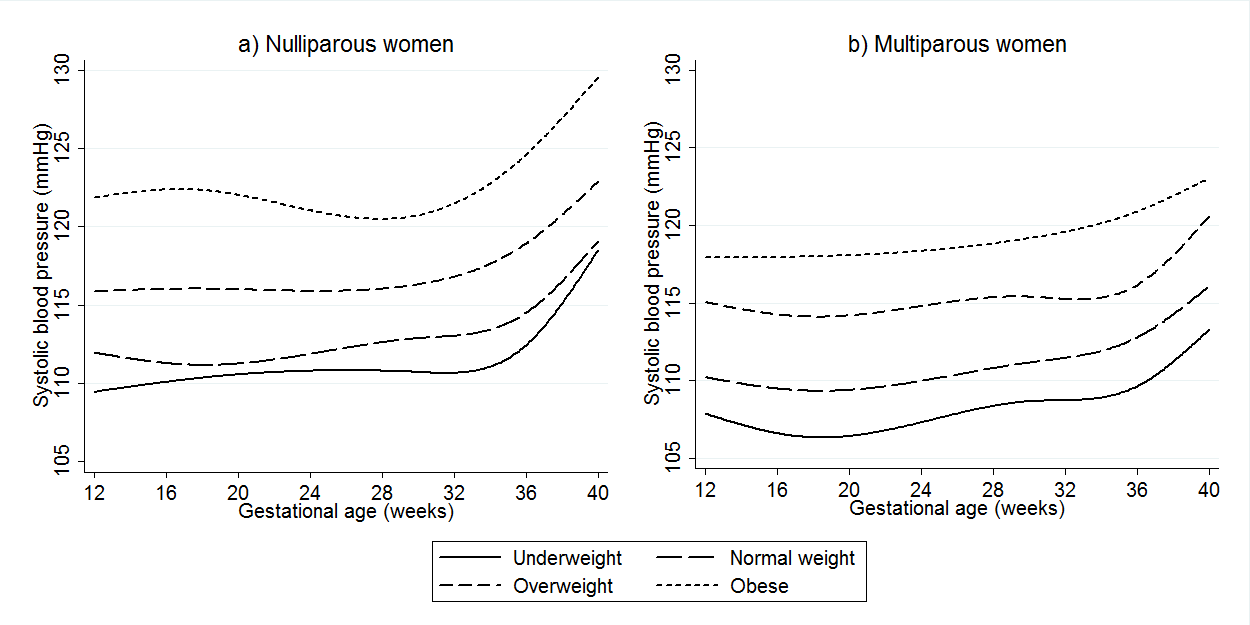
**

Web-Figure Average trajectories of diastolic blood pressure by maternal pre-pregnancy BMI category in a) nulliparous and b) multiparous non-smoking women with normal pregnancies

**
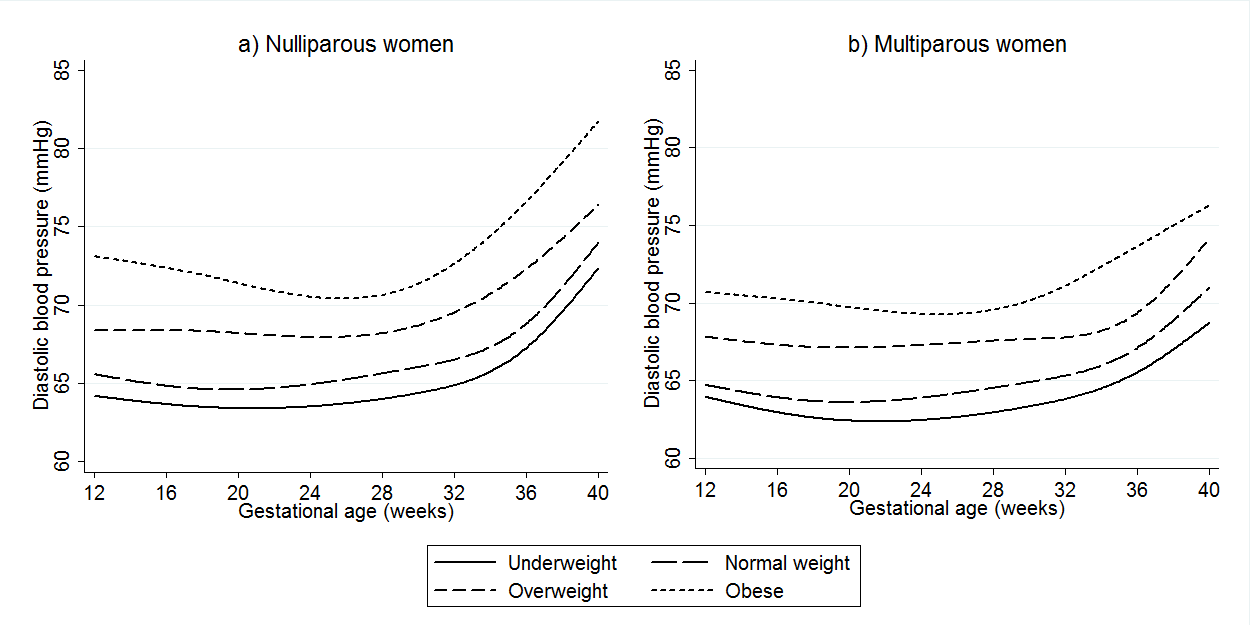
**

Web-Figure Reference ranges for systolic and diastolic blood pressure by maternal smoking during pregnancy for multiparous normal-weight women with normal pregnancies (N=3049)


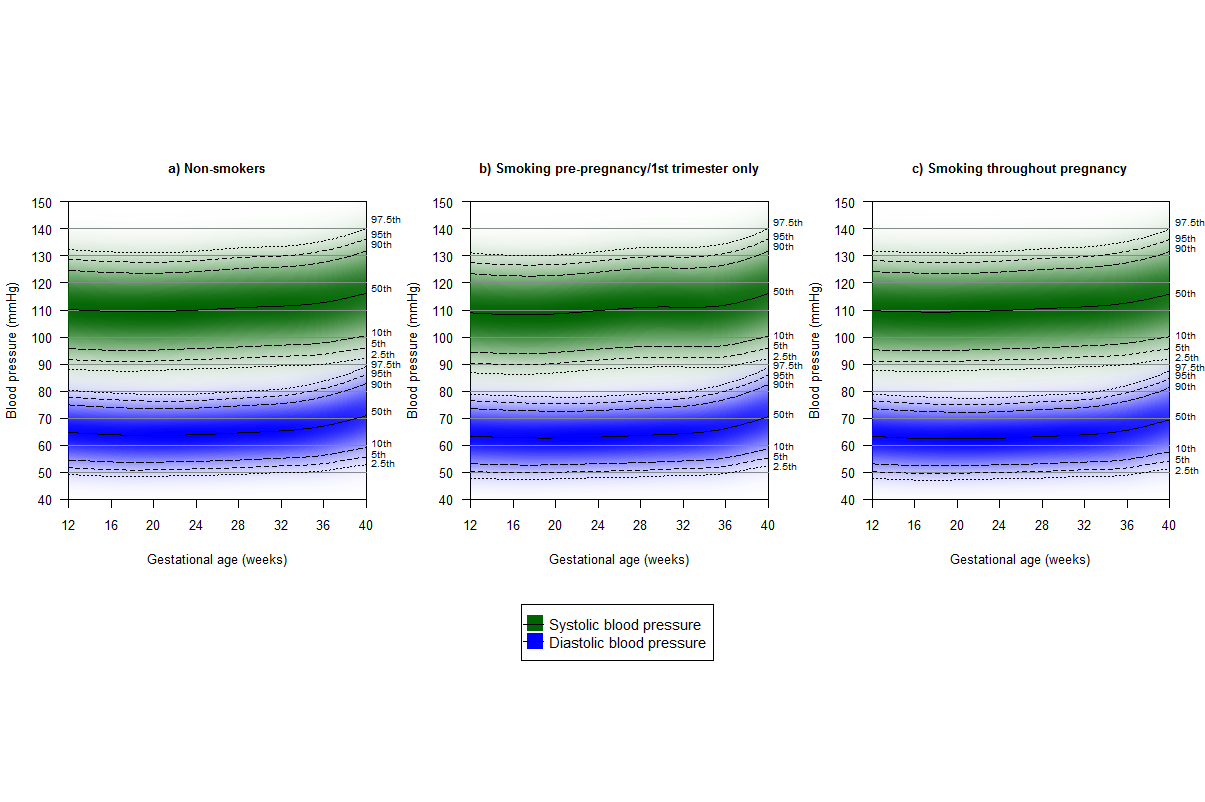


Web-Figure Average trajectories of systolic blood pressure by maternal smoking in pregnancy in a) nulliparous and b) multiparous normal weight women with normal pregnancies

**
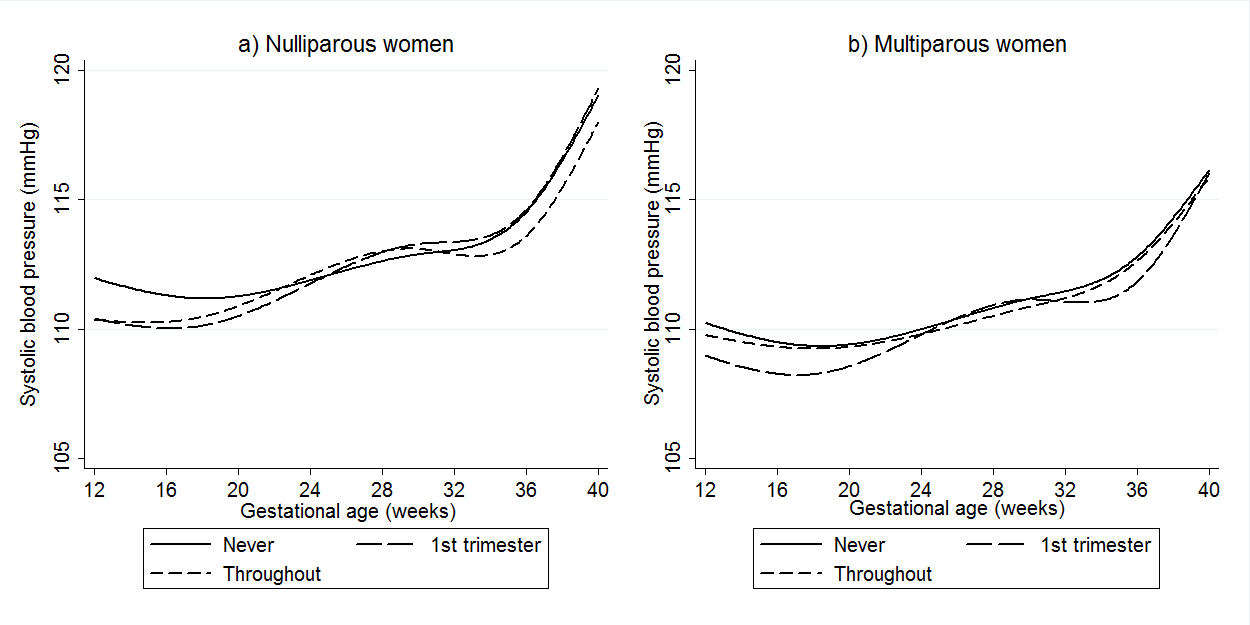
**

Web-Figure Average trajectories of diastolic blood pressure by maternal smoking in pregnancy in a) nulliparous and b) multiparous normal weight women with normal pregnancies

**
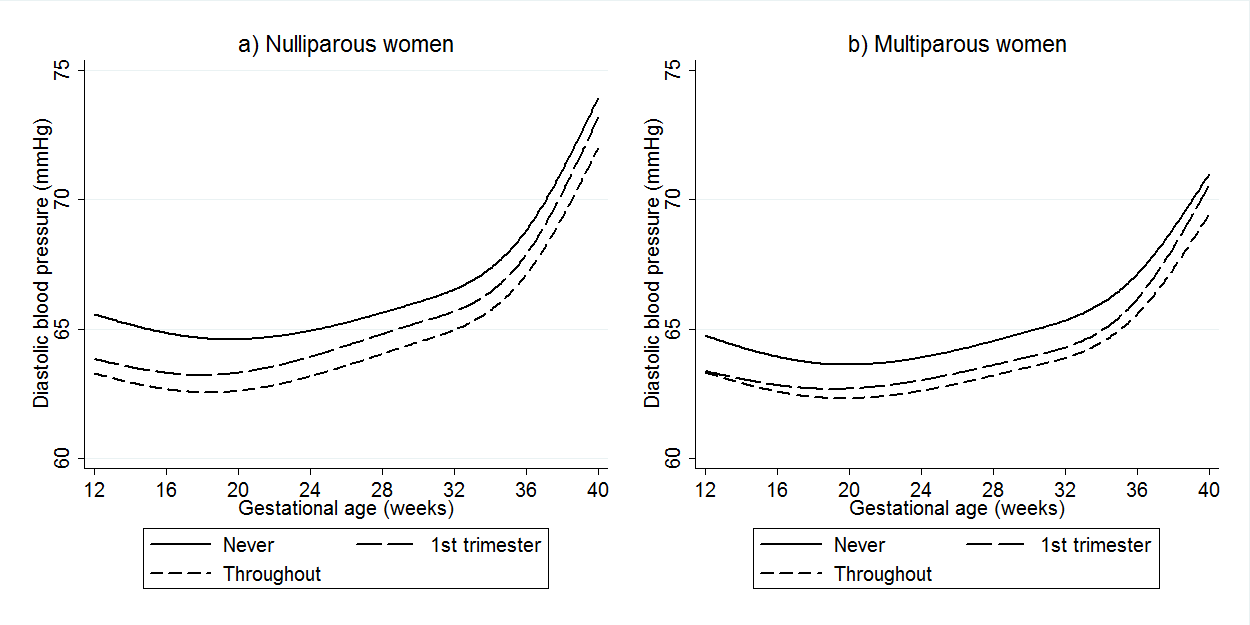
**

Web-Figure Reference ranges for systolic and diastolic blood pressure in pregnancy conditional on the level of blood pressure at 12 weeks gestation for multiparous normal-weight non-smoking women with normal pregnancies (N=2193)^*^


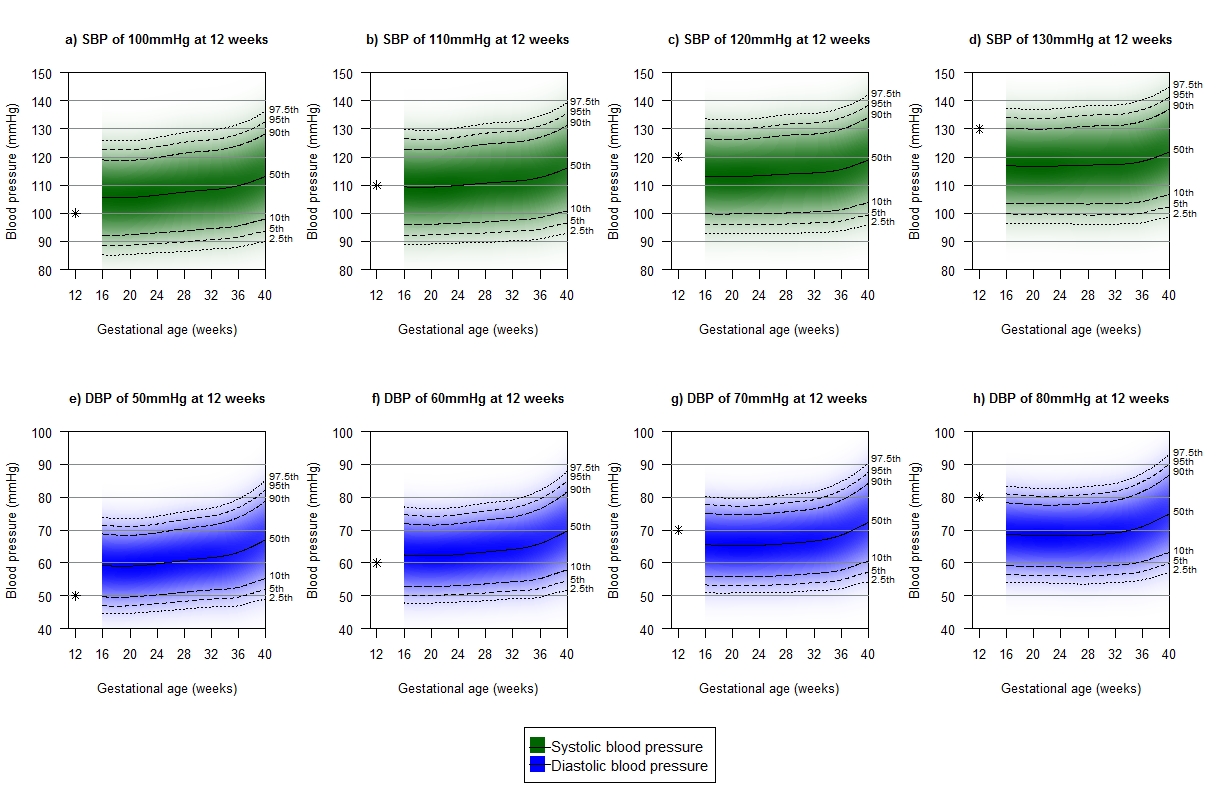


^*^ Note that in each of the plots there is a star that corresponds to the value of SBP/DBP at 12 weeks.
